# Arsenic trioxide inhibits EBV reactivation and promotes cell death in EBV-positive lymphoma cells

**DOI:** 10.1186/s12985-017-0784-7

**Published:** 2017-06-21

**Authors:** Qinyan Yin, Mark Sides, Christopher H. Parsons, Erik K. Flemington, Joseph A. Lasky

**Affiliations:** 1 Department of Medicine, Section of Pulmonary Disease, Tulane University School of Medicine, 1430 Tulane Ave, New Orleans, LA 70112 USA; 2 Department of Internal Medicine, Louisiana University School of Medicine, 1901 Perdido Street, New Orleans, LA 70112 USA; 3 Department of Pathology and Laboratory, Tulane University School of Medicine, 1430 Tulane Ave, New Orleans, LA 70112 USA; 4 Department of Internal Medicine, University of Texas Medical Branch, 300 University Blvd, Galveston, TX 77550 USA

**Keywords:** Epstein-Barr virus, EBV, Arsenic trioxide, ATO, Lymphoma, Cancer, Cancer therapy

## Abstract

**Background:**

Epstein-Barr Virus (EBV) is associated with hematopoietic malignancies, such as Burkitt’s lymphoma, post-transplantation lymphoproliferative disorder, and diffuse large B-cell lymphoma. The current approach for EBV-associated lymphoma involves chemotherapy to eradicate cancer cells, however, normal cells may be injured and organ dysfunction may occur with currently employed regimens. This research is focused on employing arsenic trioxide (ATO) as EBV-specific cancer therapy takes advantage of the fact the EBV resides within the malignant cells.

**Methods and results:**

Our research reveals that low ATO inhibits EBV gene expression and genome replication. EBV spontaneous reactivation starts as early as 6 h after re-suspending EBV-positive Mutu cells in RPMI media in the absence of ATO, however this does not occur in Mutu cells cultured with ATO. ATO’s inhibition of EBV spontaneous reactivation is dose dependent. The expression of the EBV immediate early gene Zta and early gene BMRF1 is blocked with low concentrations of ATO (0.5 nM – 2 nM) in EBV latency type I cells and EBV-infected PBMC cells. The combination of ATO and ganciclovir further diminishes EBV gene expression. ATO-mediated reduction of EBV gene expression can be rescued by co-treatment with the proteasome inhibitor MG132, indicating that ATO promotes ubiquitin conjugation and proteasomal degradation of EBV genes. Co-immunoprecipitation assays with antibodies against Zta pulls down more ubiquitin in ATO treated cell lysates. Furthermore, MG132 reverses the inhibitory effect of ATO on anti-IgM-, PMA- and TGF-β-mediated EBV reactivation. Thus, mechanistically ATO’s inhibition of EBV gene expression occurs via the ubiquitin pathway. Moreover, ATO treatment results in increased cell death in EBV-positive cells compared to EBV-negative cells, as demonstrated by both MTT and trypan blue assays. ATO-induced cell death in EBV-positive cells is dose dependent. ATO and ganciclovir in combination further enhances cell death specifically in EBV-positive cells.

**Conclusion:**

ATO-mediated inhibition of EBV lytic gene expression results in cell death selectively in EBV-positive lymphocytes, suggesting that ATO may potentially serve as a drug to treat EBV-related lymphomas in the clinical setting.

## Background

Epstein-Barr virus (EBV) is a ubiquitous DNA virus that is implicated in the pathogenesis of hematopoietic malignancies including Burkitt’s lymphoma, Hodgkin lymphoma, post-transplant lymphoma, AIDS-associated lymphomas, age-associated B-cell lymphoma, primary central nervous system lymphomas, NK/T-cell lymphoma and diffuse large B-cells lymphoma, along with non-hematopoietic tumors. EBV can establish a latent stage marked by expression of EBV latent genes (e.g. EBNA1, EBNA2, EBNA-LP, EBNA3A/3B/3C, LMP1, LMP2A/2B), and a lytic stage that expresses a set of EBV lytic genes and production of infectious virions. The switch from latent to lytic stage is driven by EBV immediate-early genes, such as BZLF1 (Zta) in vivo or by various commercial reagents in vitro, for example phorbol 12-myristate 13-acetate [1, 2], anti-IgG and anti-IgM [3–6], Ca^2+^ ionophore [7], bone morphogenetic proteins (BMPs) [8], and transforming growth factor beta 1 (TGF-β1) [9–11]. Recently, we discovered that arsenic trioxide (ATO) activates the EBV lytic cycle in nasopharyngeal carcinoma cells [12]. In general, the EBV latent cycle is associated with tumorigenesis because latent genes such as LMP1 are oncogenic, whereas the EBV lytic cycle is often considered detrimental to cell survival. However, there is evidence that the EBV lytic cycle may play a role in supporting lymphoid malignancies [13–15], in as much as patients with a higher titer of EBV lytic antigens in plasma have higher tumor recurrence rates after therapy and a poorer prognosis [16–20]. Whereas patients with lower plasma EBV DNA levels respond more favorably to current treatment regimens [21].

The mechanism by which EBV lytic genes induce malignancies has been studied but still requires clarification. The accumulated reports indicate that EBV lytic genes are directly responsible for causing malignancies and cell growth via regulation of cellular signals. Zta degrades the tumor suppressor p53 and inhibits its transcriptional function [22–26]; EBV lytic genes also inhibit antiviral cytokines such as TNF-alpha, and stimulate synthesis of cellular cytokines, such as interleukin–10, −8, and −13, which serve as growth factors to promote cell cycling and thereby tumor cell proliferation [27–29]. Moreover, induction of matrix metalloproteinases by Zta could potentially enhance metastasis of EBV-positive tumors cells via matrix degradation [30, 31]. Taken together, EBV alters cellular processes via genetic and epigenetic mechanisms, and consequently EBV-positive cell growth is dependent upon retention of the EBV genome [32–34]. Consequently, forced loss of the EBV genome in EBV-positive cells disrupts this balance and induces cell death. Studies using EBV-positive lymphoma cells have demonstrated that loss of the EBV genome in Akata cells results in cell death [35–37]. These manuscripts imply that inhibition of EBV lytic reactivation may reduce the occurrence of cancer and suggest that antiviral therapy may be useful for treating EBV-related malignancies [38].

EBV genome replication is driven by oriP during the latent phase, and by oriLyt during the lytic phase. OriLyt is located within divergent promoter regions of BHLF1 and BHRF1 and consists of two essential core elements, namely the BHLF1 promoter containing Zta response elements (ZREs) and the TD element for Sp1 binding [39]. EBV lytic DNA replication is facilitated by six core early lytic viral replication factors including: the DNA polymerase processivity factor (BMRF1 or EA-D), the primase BSLF2, the helicase BBLF4, the helicase-primase complex BBLF2/3, the single-stranded DNA-binding protein BALF2 and the DNA polymerase BALF5 [40–43]. Importantly, BZLF1 is a central regulator for lytic replication, binding directly to ZRE sites on the upstream domain of oriLyt and interacting with other viral core replication proteins, such as BMRF1, BALF2 and BBLF4 [44–46].

ATO is a highly effective in treating acute promyelocytic leukemia [47] via degradation of promyelocytic leukemia (PML) nuclear bodies through a ubiquitin-mediated pathway [48–51]. ATO treatment results in degradation of all 7 major PML isoforms, in which PMLV is the most highly degraded isoform and PMLIV is the least degraded isoform [52]. ATO binds to PML-RARα protein directly and induces its SUMO modification by recruiting a ring-domain-containing ubiquitin E3 ligase (RNF4), along with ubiquitin and the proteasome, to PML nuclear bodies, resulting in the degradation of PML-RARα [53, 54]. EBV latent proteins (LMP2A, LMP1 and EBNA1) interact with the cellular proteasome/ubiquitin pathway to control the EBV latency. However, EBV lytic proteins, such as Zta, Rta and BMRF1, can also be regulated by sumoylation and ubiquitination. Zta is a bZIP protein that can be SUMO1 modified on Lys 12 and Lys15 [55, 56]. Rta can be sumoylated on Lys-19, Lys-213, and Lys-517 [57]. SUMO-targeted RNF4 interacts with Rta and enhances Rta and BMRF1 ubiquitination [58]. Overexpression of Zta in EBV latently infected cells results in dispersion of PML nuclear bodies and induces loss of SUMO1-modified isoforms of PML protein [59, 60]. Knockdown of PML reduces the production of viral particles and EBV genome in EBV-positive P3HR1 cells, supporting the concept that PML nuclear bodies play a role in EBV capsid assembly and viral lytic DNA replication [61].

We previously demonstrated that ATO activates the EBV lytic cycle in EBV-positive epithelial cells and inhibits tumor growth in a xenograft model [12]. In contrast, in this manuscript we did not find that arsenic induced EBV reactivation in Burkitt’s lymphoma cells. In the work presented here we show that arsenic inhibits the expression of EBV lytic genes Zta, Rta and BMRF1, and promotes cell death in EBV-positive lymphoma cells. Herein, we also report that ATO regulates EBV reactivation via ubiquitin/proteasome-dependent proteolysis. Current therapies for anti-EBV-positive lymphomas are not vastly different in comparison to EBV-negative lymphomas. We submit that arsenic may be a potential antiviral chemotherapy for treatment of EBV-associated lymphomas.

## Methods

### Cell culture and treatment

EBV-positive latency type I Burkitt’s lymphoma cell lines (Mutu, Akata, BX-1, Rael and SAV5), an EBV latency type II B lymphocyte cell line (Cl13), and EBV latency type III lymphoblastoid cells (JY), have been maintained in our laboratory for more than 20 years through freeze-thaw cycles. The Farage EBV-positive diffuse large B-cell lymphoma cell line was purchased from ATCC. PBMC cells were a gift from Dr. Frédéric Ganapamo. Cell culture conditions were as described previously [62]. Briefly, all cell lines were cultured in RPMI 1640 media supplemented with 10% heat-inactivated fetal bovine serum (Gibco) in a humidified incubator with 5% CO_2_ at 37 °C. The cells were split 1:1 1 day before treatment. On the day of treatment, cells were enumerated and viability assessed using trypan blue exclusion staining. Cells were re-suspended with fresh media at the confluence of 1 × 10^6^ cells per ml, and ATO was added at the indicated concentrations. For proteinase or sumoylation inhibitor experiments, MG132 or Ginkgolic Acid respectively were added 4 h or 16 h prior to harvest. ATO (Sigma # A1010) was dissolved in 1 M NaOH and stored at −20 °C as a 250 mM stock solution. The 1 μM working solution was prepared by dilution in sterile PBS (arsenic is adherent to commercial cell culture filters, so such filters were not employed). Ganciclovir was dissolved in 0.1 N HCl at a concentration of 10 mg/ml and stored at −20 °C.

### Western blotting and immunoprecipitation

Cells were lysed with RIPA buffer (Cell Signaling) supplemented with 0.1 M phenylmethyl sulfonyl fluoride (PMSF), protease inhibitor mixture, and phosphatase inhibitors 2 & 3 (Sigma)). Protein concentrations for western blotting and immunoprecipitation were determined using the Bio-Rad protein assay reagent and a Beckman Coulter spectrophotometer. Immunoprecipitation experiments were conducted as described previously [63]. Briefly, 500 μg of protein was used for each immunoprecipitation and precleared with 50 μl protein A/G sepharose beads (Santa Cruz) for 6 h. Antibody (2 μg) was incubated with 20 μl protein A/G sepharose beads for 6 h to overnight. The immune-complexes were washed 3 times with RIPA buffer before being resolved using 2× SDS-PAGE loading buffer (sigma) and separated on a 4–20% Tris-HCl gradient SDS-PAGE gel (BioRad). The signal was detected using the Odyssey Infrared Imaging System (Li-Cor Biosciences). The following antibodies were used: Actin (sc-1616), Zta (Argene 11–007), Rta (Argene 11–008), BMRF1 (EBV-018-48,180), BGLF4 (Argent AP8057b), VCA (Argene 11–019), LMP1 (BD 559898) and GAPDH (Cell Signaling 2118 L).

### RNA extraction and quantitative reverse transcription (RT)-PCR

Total cellular RNA was isolated using the RNeasy Plus mini kit (Qiagen #74136) and was reverse transcribed using the iScript cDNA Synthesis Kit (BioRad, Cat# 170–8890). The expression level of EBV genes, Zta and LMP1 were determined by SYBR green dye chemistry and calculated using the 2^–∆∆*C*T^ method. Primers used for RT-PCR: LMP1 forward: 5′-CTACTGATGATCACCCTCCT-3′ and reverse: 5′-ATACCGAAGACAAGTAAGCA-3′; Zta forward: 5′ GGGGGATAATGGAGTCAACA 3′ and reverse: 5′ GGAAACCACAACAGCCAGAA 3′; 36B4 forward: 5′- CGAGGTGGAAGTCCAAGT-3′ and reverse: 5′-ATGTGGTGCATCTGGTTG-3′.

### Cellular and viral DNA extraction and quantitative real-time PCR

Total cellular DNA was isolated with DNAzol (Invitrogen #10503–027) and was quantified using the nanodrop method. Four ng of DNA was used for real-time PCR in a 20 μl volume. Viral DNA was isolated from media as described previously [63]. Briefly, cell culture media was filtrated through a 0.45 μm SFCA filter and incubated with proteinase K (Invitrogen) at 37 °C for 1 h to overnight, followed by incubation at 95 °C for 15 min prior to extraction using phenol/chloroform. 100× diluted media extraction was used for real-time PCR with primers spanning the BamHI Z region and Q promoter (Qp) regions of the EBV genome and housekeeping gene GAPDH. The quantitative level was calculated using the 2^–∆∆*C*T^ method. Primers used for PCR were: BamHI Z forward: 5′-TTGACACCAGCTTATTTTAGACACTTCT-3 and reverse: 5′-TTACCTGTCTAACATCTCCCCTTTAAA-3′; Qp forward: 5′-AAATTGGGTGACCACTGAGG-3′ and reverse: 5′-CATACACCGTGCGAAAAGAA-3′; GAPDH Forward: 5′-AAGGTGAAGGTCGGAGTCAAC-3′ and reverse 5′-GGGGTCATTGATGGCAACAATA-3′.

### MTT assay and cell viability

The MTT assay was performed following the manufacturer’s instructions (Sigma #M-8910). Briefly, cells were cultured in phenol free medium and an equal volume of reconstituted MTT was added onto the cells. The MTT solubilization solution was added and mitochondrial dehydrogenases activity was measured using a microplate auto reader (Bio-tek Instruments) after 2–4 h of incubation. Cell viability was measured by enumeration using a trypan blue (Invitrogen) method.

### Statistical analysis

Statistical significance of each variable was assessed using one-way ANOVA.

## Results

### ATO inhibits EBV lytic gene expression and genome replication

#### ATO inhibits EBV lytic gene expression

**Fig. 1:**
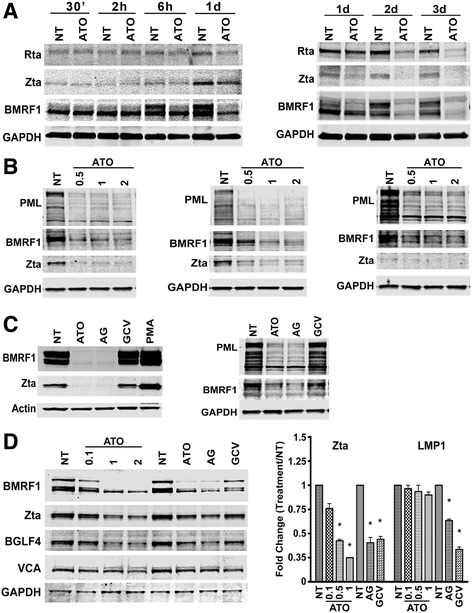
ATO inhibited EBV reactivation at low concentrations in EBV-positive lymphoma cells. **a** ATO inhibited EBV spontaneous reactivation in Mutu cells. Western blotting detected EBV spontaneous reactivation within 6 h after re-suspending cells, but not in cells cultured in the presence of 1 nM of ATO. **b** ATO inhibited EBV lytic gene expression in a dose-dependent manner. EBV latency type I cells (*from left to right*: Mutu, Rael and PBMC) were treated with various concentrations of ATO (0.5 nM – 2 nM) for 3 days and harvested for western blotting. **c** Co-treatment with ATO and GCV (AG) inhibits EBV lytic gene expression. Western blotting assessment of EBV lytic gene expression in Rael (*left*) and SAV5 (*right*) after treatment with 1 nM ATO and/or 45 μM GCV for 3 days. AG indicates treatment with ATO plus GCV. **d** ATO inhibited EBV gene expression at both the RNA and protein levels. Mutu cells were treated with ATO at the indicated concentration for 3 days or with 0.5 nM ATO with/without 45 μM GCV and harvested for western blotting and real-time RT-PCR. ^*^ *p* < 0.05 vs no treatment (NT)

EBV-positive latency type I cell Mutu cells displayed spontaneous reactivation after being re-suspended in fresh media for 6 h, and reached maximal lytic cycle by day 1 (Fig. 1a left). The EBV lytic gene BMRF1 and immediate early gene Zta were induced as early as 6 h after resuspension in fresh media. In contrast, BMRF1 was not induced when the media contained ATO, and the expression of Zta and Rta were lower compared with no treatment (NT) at day 1, 2 and 3 (Fig. 1a right). ATO-mediated EBV inhibition was dose-dependent (Fig. 1b). ATO inhibited expression of the EBV lytic genes BMRF1 and Zta in EBV latency type I cells, Mutu (Fig. 1b left) and Rael (Fig. 1b middle), when treated with ATO for 3 days at the indicated concentrations. PML protein levels were reduced by ATO as expected. In addition, ATO inhibited EBV gene and PML expression in peripheral blood mononuclear cells (PBMC) infected with EBV in vitro (Fig. 1b right).

Co-treatment of ATO with ganciclovir (GCV), a virus DNA replication inhibitor, decreased PML protein expression as well as that of the EBV BMRF1 in EBV-positive latency type I cells, Rael (Fig. 1c left) and SAV5 (Fig. 1c right). PMA was used as a positive control to induce the expression of the EBV lytic genes BMRF1 and Zta in Rael cells.

Mutu cells treated with various concentrations of ATO (0.1– 2 nM) for 3 days were harvested for western blotting and RT-PCR. ATO (0.1 nM) inhibited the expression of the EBV immediate early gene Zta, along with other lytic genes including BMRF1, BGLF1 and VCA (Fig. 1d left). ATO treatment did not affect the mRNA level of the EBV latent gene LMP1. Zta mRNA expression was inhibited by ATO at 0.5 nM and 1 nM (Fig. 1d right). In contrast, the combination of ATO and ganciclovir blocked the expression of LMP1 mRNA.

#### ATO inhibits EBV genome replication

**Fig. 2:**
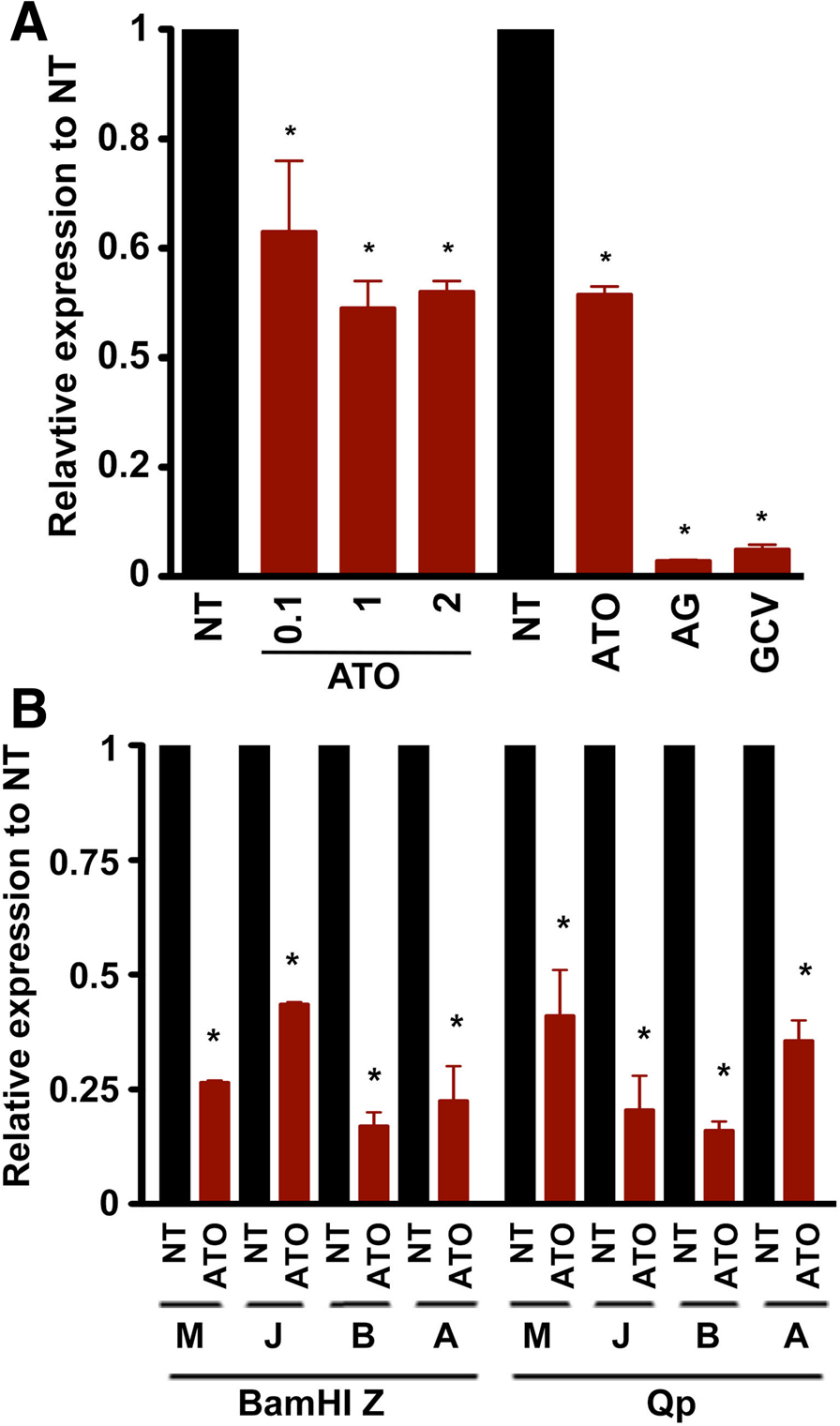
ATO inhibits EBV genomic DNA replication. **a** Mutu cells were treated with 1 nM of ATO for 3 days and cell media was harvested for viral genome extraction. PCR was performed using primers spanning the BamHI Z fragment of the EBV genome, ^*^ *p* < 0.05 vs. NT. **b** EBV-positive cells, Mutu (M), JY (J), BX-1(B), and Akata (A) cells were treated with 1 nM of ATO for 5 days and cellular total DNA was extracted for PCR using primers spanning the BamHI Z region and the Qp region of EBV genome, * *p* < 0.05 vs. NT. The relative expression of BamHI Z and Qp was calculated using the comparative Ct method (2^–∆∆*C*T^)

Downregulation of EBV lytic gene expression by ATO prompted investigation into the effects of ATO on EBV replication. To quantify EBV viral load, viral DNA was extracted from the media, in which the cells were cultured. Real-time PCR was performed to assess genomic DNA levels through detection of a region encompassing the EBV BamHI Z fragment. The viral genome level was low in ATO-treated cell media compared to untreated cell media, and co-treatment of ATO with GCV blocked the genome level even more dramatically (Fig. 2a). GCV alone also inhibited EBV genome levels. We also extracted total cellular DNA and viral DNA from ATO-treated EBV-positive Mutu (M), JY (J), BX-1(B) and Akata (A) cells (Fig. 2b) and quantified the viral genomic DNA (BamHI Z fragment and a region surrounding the EBNA1 Qp promoter). ATO inhibited viral DNA genome accumulation, indicating that ATO blocked EBV genome replication or decreased the EBV genomic DNA levels.

### ATO promotes greater cell death in EBV-positive cells than in EBV-negative cells

#### ATO-induced cell death is dose- and time-dependent

**Fig. 3:**
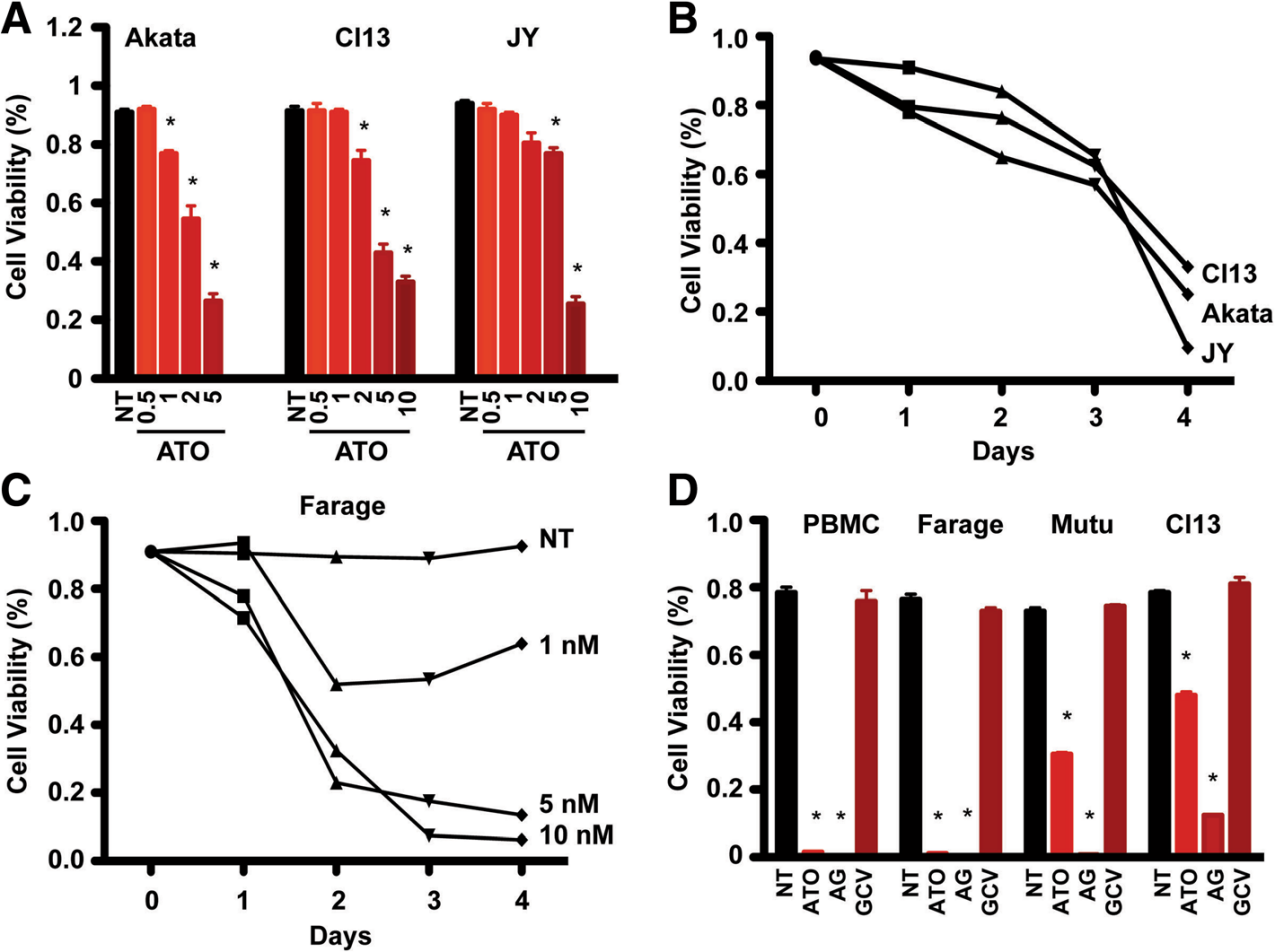
ATO inhibited cell viability in a dose- and time-dependent manner. **a** EBV latency type I (Akata), type II (Cl13) and type III (JY) cells were treated with ATO for 3 days at the indicated concentrations (0.5 nM – 10 nM) and cell viability was analyzed using a trypan blue method. *, *p* < 0.05 vs. no treatment (NT). **b** The time course analysis of cell viability in response to 2 nM of ATO in EBV-positive cells. **c** The dose- and time-course analysis of cell viability after treatment with ATO in Farage cells. **d** The combination of ATO (10 nM) and ganciclovir (45 μM) synergistically decreased cell viability on day 3 in EBV-positive cells. ^*^, *p* < 0.05 vs. NT

To test the influence of ATO on cell viability, EBV-positive latency type I Akata, latency type II Cl13, and latency type III JY cells were treated with ATO at the indicated concentrations (0.5 nM – 10 nM). Cell viability was measured daily via the trypan blue method. ATO decreased cell viability in a dosed-dependent manner (Fig. 3a). ATO induced cell death at 1 nM in Akata and 2 nM in Cl13 cells and 5 nM in JY cells on day 3. Furthermore, the decrease in cell viability was dependent on the duration of ATO exposure. As shown in Fig. 3b, cell viability decreases by 15–30% at day 2, but by day 4 viability was almost entirely lost.

To determine the viability of diffuse large B-cell lymphoma cells in response to ATO, diffuse large B-cell lymphoma cells (Farage) were treated with ATO at the indicated concentrations and cell viability was determined using trypan blue (Fig. 3c). Farage cell viability was decreased by approximately 50% after 2 days of treatment with 1 nM of ATO, and was diminished further at a higher concentration (10 nM) and longer treatment duration (4 days).

We did not observe any difference in cell viability in response to ganciclovir alone, indicating that ganciclovir cannot induce cell death in EBV-positive PBMC cells, diffuse large B-cell lymphoma cells (Farage), or other lymphoma cell lines (Mutu and Cl13) (Fig. 3d). Nevertheless, the combination of ganciclovir with ATO significantly decreased cell viability and induced much greater cell death compared the effect of ATO alone. ATO treatment resulted in a 50–70% loss of cell viability compared to no treatment in CL13 and Mutu cells respectively and this effect was even more pronounced, specifically a 90% loss in cell viability, when ATO and ganciclovir were employed together for 3 days. PBMC and Farage cells also demonstrated enhanced sensitivity to the combination of ATO and ganciclovir. Viability was minimal in PBMC and Farage cells after 3 days of treatment with 10 nM of ATO, alone or with ganciclovir.

#### ATO specifically decreases EBV-positive cell viability and cell growth

**Fig. 4:**
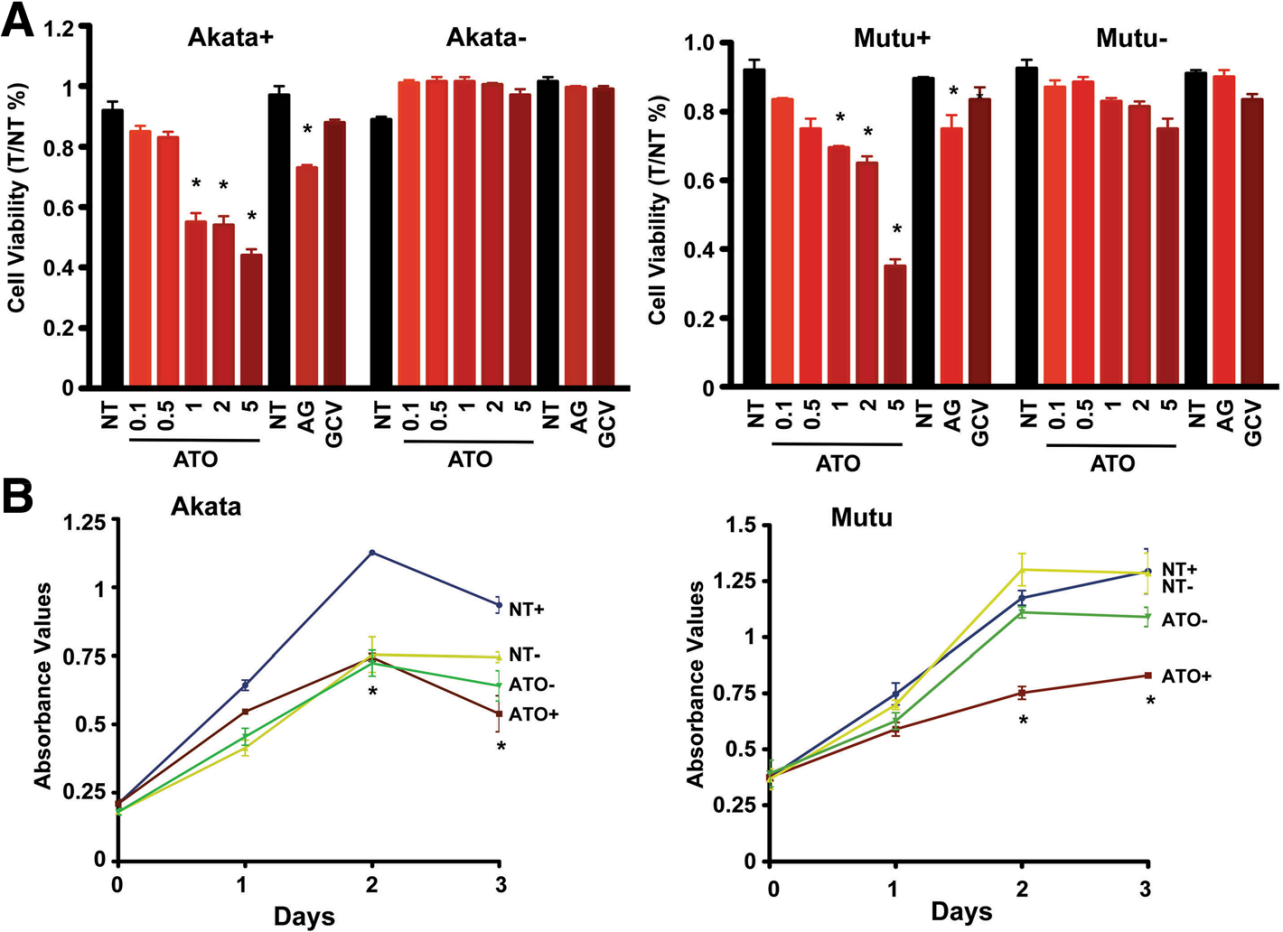
ATO decreased cell viability and growth in EBV-positive cells but not in EBV-negative cells. **a** EBV-positive cells (Mutu+, Akata+) and EBV-negative cells (Mutu-, Akata-) were treated with ATO alone at the indicated concentrations (0.1 nM – 5 nM) or with 45 μM of ganciclovir (GCV) for 3 days and cell viability was determined using trypan blue (AG indicates the combination of 0.5 nM of ATO and 45 μM of GCV). * *p* < 0.05 vs no treatment (NT). **b** Mutu and Akata cells were treated with 1 nM of ATO and a MTT assay was performed daily for 3 days (+ represents EBV-positive cells, − indicates EBV-negative cells, NT denote no treatment). ^*^ *p* < 0.05 vs NT

In this set of experiments, we assessed whether loss of cell viability with ATO or ATO/GCV is specific to EBV-positive lymphoma cells. EBV-positive Mutu (Mutu+)/ Akata (Akata+) and EBV-negative Mutu (Mutu-) / Akata (Akata-) cells were treated with ATO at the indicated concentrations for 3 days and cell viability was measured. As shown in Fig. 4a, cell viability decreased from 90% viable cells to 53% viable cells in Akata + cells and from 92% viable cells to 65% viable cells in Mutu + cells at a 1 nM concentration of ATO. Higher concentrations of ATO (5 nM) decreased viability to 35 and 41% in Akata + and Mutu + respectively. Time course experiments employing an MTT assay demonstrated that ATO (1 nM) decreased EBV-positive cell proliferation at day 1, which was also evident on day 3 (Fig. 4b). Compared to untreated cells, EBV-positive Akata cell growth was decreased by 16–42%, and Mutu cell growth decreased by 20–38%. In contrast, EBV-negative cell growth showed no significant change compared to untreated cells (Fig. 4b).

### ATO inhibits EBV reactivation through ubiquitination and sumoylation pathways

**Fig. 5:**
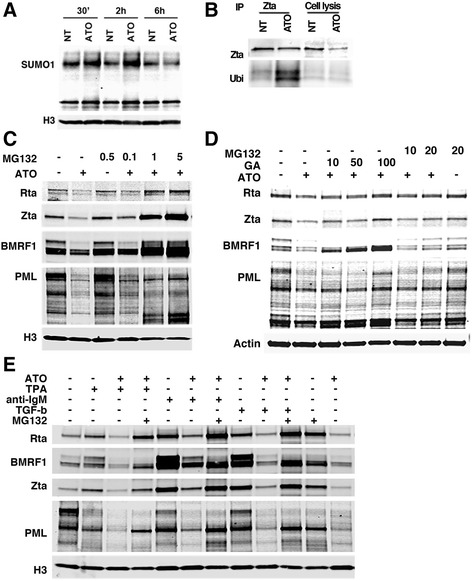
EBV lytic proteins were ubiquitined and sumoylated in response to ATO treatment and this effect was rescued using proteasome and SUMO inhibitors in EBV latency type I Mutu cells. **a** 1 nM of ATO induced SUMO1 expression. **b** Co-IP with antibodies against Zta and western blotting using antibodies against ubiquitin. **c** MG132 at various concentrations (0.1 μM – 5 μM for 16 h) rescued EBV spontaneous reactivation that was reduced in response to 1 nM of ATO. **d** Proteasome (MG132) & SUMO1 (Ginkgolic Acid, GA) inhibitors rescued the reduction in EBV reactivation in response to ATO. Cells were treated with 1 nM of ATO for 3 days and with/without MG132 or GA at indicated concentrations (μM) for 4 h. **e** 1 nM ATO inhibited the anti-IgM-, PMA-, and TGF-β-induced EBV reactivation and MG132 (1 μM for 16 h) abrogated this effect

We sought the mechanism through which ATO inhibits EBV gene expression. It is established that ATO degrades PML through SUMO-mediated ubiquitination [53, 54, 64]. To determine whether ATO inhibits EBV reactivation via the ubiquitin-mediated pathway, SUMO1 expression was assessed in response to ATO treatment in EBV-positive lymphoma cells. SUMO1 was increased at early time points, specifically 30 min and 2 h after ATO treatment, indicating that ATO may promote EBV protein degradation through the SUMO1-induced ubiquitin pathway (Fig. 5a). To further investigate this, the EBV gene Zta was immunoprecipitated from these samples and was observed to co-precipitate of ubiquitin. As shown in Fig. 5b, ubiquitin protein levels were higher in ATO-treated samples, indicating that ATO induced Zta-bound ubiquitin, and that Zta is ubiquitinated, which fosters its degradation. Taken together, these data suggest that ATO inhibits EBV protein expression via ubiquitin-mediated protein degradation.

To further test this hypothesis, inhibition experiments to block protein ubiquitination and sumoylation were performed by treating cells with the proteasome inhibitor MG132 and the protein sumoylation inhibitor Ginkgolic acid (GA). MG132 rescued ATO-mediated PML degradation as well as ATO-induced EBV protein degradation (Fig. 5c and d). PML protein expression was inhibited by ATO (1 nM) and MG132 rescued PML expression (0.1 μM for 16 h (Fig. 5c) or 20 μM for 4 h (Fig. 5d)). Interestingly, MG132 also recovered ATO-mediated inhibition of EBV protein expression, specifically Zta, Rta and BMRF1 (Fig. 5c and d). MG132 alone did not affect EBV gene expression at 0.5 μM for 16 h, implying that MG132 mediates EBV recovery from ATO through inhibiting EBV protein ubiquitination. Thus, ATO’s effect on EBV protein expression is mediated through the proteasome ubiquitin pathway. To assess whether ATO-induced sumoylation is involved in EBV protein ubiquitination, cells were treated with ATO along with the protein sumoylation inhibitor Ginkgolic acid (Fig. 5d). EBV genes Zta, Rta and BMRF1, and the cellular protein PML, was inhibited by ATO, and notably inactivation of sumoylation using 10 μM Ginkgolic acid for 4 h completely blocked their degradation. These observations provide supportive evidence that both sumoylation and ubiquitination contribute to ATO-induced EBV protein degradation.

To better understand the ATO-induced EBV lytic protein degradation pathway, a series of combination treatments were performed in EBV-positive latency type I cells. EBV reactivation by BCR signaling (anti-IgM), protein kinase C activation [1] and TGF-β signaling provide unique models to investigate ATO-mediated EBV protein degradation. First, a combination of ATO with each of these signaling reagents was applied to cells, and the proteasome inhibitor MG132 was added prior to harvest. As shown in Fig. 5e, ATO inhibited anti-IgM-, PMA- and TGF-β-mediated EBV reactivation. The expression of Zta, Rta and BMRF1 were activated by anti-IgM, PMA or TGF-β, and inhibited after co-treatment with ATO. Importantly, MG132 rescued the ATO-mediated inhibition of EBV reactivation induced by anti-IgM, PMA or TGF-β (Fig. 5e). Therefore, ATO disrupts the EBV infection cycle and inhibits EBV gene expression through activation of global cellular protein ubiquitination. These results indicate that ATO induces the rapid sumoylation of Zta, Rta and BMRF1, resulting in their ubiquitination and proteasome-dependent degradation. Thus, ATO-mediated EBV protein degradation is dependent on SUMO-regulated protein ubiquitination and proteasome-mediated degradation.

## Discussion

We have shown that ATO inhibits EBV reactivation through ubiquitin-mediated degradation. The consequences of this were inhibition of EBV replication and induction of cell death in EBV-positive cells. This result is consistent with a previous report that loss of the EBV genome and lytic gene expression leads to the loss of the malignant phenotype and cell viability in EBV-positive Burkitt’s lymphoma cells [65–67]. EBV lytic genes are expressed in 29% of lymphoma patients according to Dr. Liu’s report [67]. In EBV-positive cells, the lytic viral proteins regulate diverse homeostatic cellular functions including inflammation and angiogenesis. Thus, the small portion of cells in the lytic cycle may support tumor cell growth and survival by providing cell growth factors and other signals. Diminishing lytic gene expression in cells exposed to ATO eradicates EBV genome replication and results EBV-dependent cell death.

Spontaneous EBV reactivation provides us a cell system to evaluate the anti-tumor effect of ATO on lymphoma cells. The expression of EBV lytic genes is decreased significantly in response to ATO treatment, suggesting that ATO promotes EBV lytic protein degradation (Fig. 1). Further, the growth rate in cell populations with spontaneous EBV reactivation is faster than in cells treated with ATO. More importantly, inhibiting EBV lytic gene expression in cells exposed to ATO impedes the proliferation of these cells (Figs. 3 & 4), but this was not observed in EBV-negative cells. In EBV-associated lymphomas and other tumors, spontaneous reactivation is thought to play an important oncogenic role [67, 68], and using reagents to inhibit EBV lytic gene expression reduces EBV-positive cell viability [67]. The above evidence adds support to the idea that ATO could serve a therapeutic agent for EBV-positive lymphomas.

On the other hand, lytic induction by reagents in vitro eventually leads to a more persistent latent stage, which induces other oncogenes that may foster the development of malignancies. Thus, the major concern is that lytic induction by chemotherapy is also followed by stages of latency. Treatment of Akata and Mutu cells with anti-IgG or anti IgM induces latent gene expression [69]. Moreover, Akata cells remain viable much longer after treatment. Also, co-expression of lytic replication and latency proteins has been detected in vivo [70, 71]. Though lytic induction therapy looks promising, its toxicity and side effects cannot be avoided, and it may lead to more persistent latent infection. Thus, the combination of ATO with lytic inducers is a possible alternative strategy for anti-EBV associated lymphomas.

The presence of EBV lytic cycle replication in these cells promotes the expression of BGLF4 (Fig. 1d), which is a viral-encoded protein kinase that phosphorylates ganciclovir to its active form (monophosphorylated) [72]. The phosphorylated ganciclovir incorporates with viral and cellular DNA and kills the cells via disrupting replication [73], providing a plausible explanation for the diminished EBV gene expression in response to ganciclovir. However, due to the low level of BGLF4 necessary for converting the ganciclovir into its active form, it cannot independently induce significant cell death.

The circular EBV chromosome replicates once with each cell division and depends on cellular replication machinery during the latent stage [74], whereas EBV-encoded lytic genes drive EBV lytic replication and yields numerous copies of viral genomes within each cell. As a lytic transactivator, Zta binds to the lytic replication origins (oriLyt) and activates the other EBV lytic genes to initiate EBV lytic replication. Zta recruits the EBV core replication machinery and other cellular proteins into the oriLyt region to initiates EBV lytic replication after Zta binds to EBV oriLyt. Thus, inhibition of Zta or other related EBV lytic replication factors would eradicate the EBV episomal genome. Moreover, as a transcription activator, Zta is required for the transcriptional activation of its own promoter BZLF1 [75], as well as the promoters of other lytic genes such as BRLF1, BMRF1 and BALF2 (the major DNA-binding proteins) [3, 76–78]. Thus, reduction of Zta expression would be expected to reduce Rta, BMRF1 and BALF2 expression, and further diminish Zta expression. Hence, inhibition of Zta expression or Zta transcriptional activity decreases not only EBV lytic gene expression but also the production of the EBV episomal genome. This explains why EBV gene expression and the EBV genome are decreased after ATO treatment as shown in Figs. 1 & 2.

Arsenic activates EBV reactivation in epithelial cells (NPC-KT) [12], but inhibits EBV lytic genes expression in lymphoma cells. This may be due to differences between anchored epithelia cells in comparison to lymphoma cells in suspension. Cell signaling is different between these two cell types. For example, the oncoprotein c-Myc is over-expressed and translocated in most lymphomas. C-Myc not only regulates cell biological function but is also involved with sumoylation regulators, such as SUMO2/3 and E1/2/3 ligases [79], which are in turn regulated by arsenic and contribute to degradation of EBV lytic gene expression in lymphoma cells. In contrast, NPC-KT cells are EBV latency type II cells that express the EBV latent genes LMP2A and LMP1, which can interact with the ubiquitin/proteasome system to regulate gene expression [80, 81]. These interactions could interfere with arsenic’s modulation of the ubiquitin pathway and curb arsenic’s effects on EBV lytic gene expression.

**Fig. 6:**
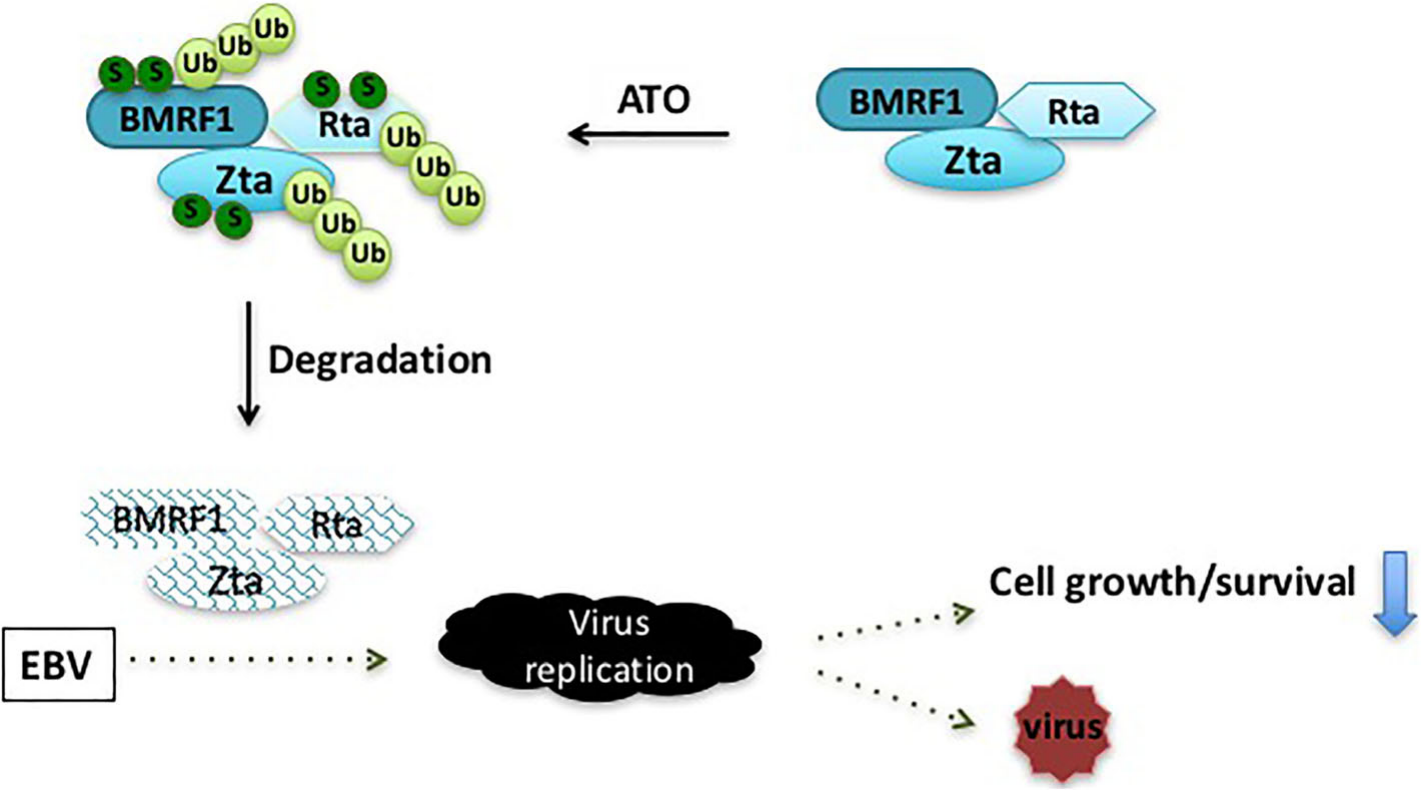
A depiction of how ATO regulates the EBV lytic cycle and cell fate in EBV-positive lymphoma cells. Exposure to ATO induces EBV lytic protein degradation through sumoylation and ubiquitination. Consequent to their degradation, EBV lytic genes cannot activate EBV lytic replication, which in turn diminishes signaling required for cell growth. Ultimately ATO leads to cell death in EBV-positive cells

Our results show that ATO not only blocks EBV spontaneous reactivation but also reagent-induced reactivation (Fig. 5e), implying that ATO-inhibited EBV lytic gene expression occurs through a broadly utilized pathway. Proteasome or sumoylation inhibitors rescue the ATO-mediated reduction of EBV reactivation in a dose dependent manner. Furthermore, the co-immunoprecipitation experiment reveals that ATO leads to greater ubiquitinization of the Zta protein. Thus, these results indicate that ATO induces EBV lytic protein ubiquitination and proteasome-mediated degradation, and that sumoylation may facilitate the degradation process. On the basis of our observations, we propose the molecular mechanistic model for arsenic-mediated degradation of EBV lytic genes and cell death in EBV-positive lymphoma cells illustrated in Fig. 6. Lytic gene expression will lead to cellular protein expression that provides signals for cell growth and tumorigenesis. In the presence of ATO, spontaneous and reagent-induced EBV reactivation is abolished, and involves decreased expression of EBV lytic genes by degradation of Zta, Rta and BMRF1 via sumoylation and ubiquitination. As a result, EBV cannot provide sufficient cell survival factors and results to cell death.

## Conclusions

Most antiviral drugs have limited efficacy for treating EBV-related malignancies. ATO has received prior recognition as a cancer therapy due to its effectiveness in treating acute promyelocytic leukemia [47]. Several studies have shown that ATO may be useful for the treatment of other cancers such as ovarian, brain, breast, lung, gastric and cervical cancers [82–87]. However, its potential for the treatment of lymphoma has not been previously advanced. Our data suggests that ATO may be an effective therapeutic drug for EBV-specific lymphomas. We believe the mechanism by which ATO induces EBV gene ubiquitination and degradation requires further investigation, and in vivo murine tumor xenograft experiments will be prudent prior to clinic trials in humans.
